# Ceftazidime-avibactam plus aztreonam cocktail for the treatment of VIM-producing *Pseudomonas aeruginosa* infections: good enough to have another?

**DOI:** 10.1093/jac/dkaf083

**Published:** 2025-03-19

**Authors:** Manuel Pina-Sánchez, Marta Rua, Carla López-Causapé, Idoia Bilbao, Miquel Àngel Sastre-Femenia, Antonio Oliver, José Luis Del Pozo

**Affiliations:** Service of Clinical Microbiology, Clínica Universidad de Navarra, Av. De Pio XII, 36, Pamplona, Navarre, 31008, Spain; Service of Clinical Microbiology, Clínica Universidad de Navarra, Av. De Pio XII, 36, Pamplona, Navarre, 31008, Spain; Instituto de Investigación Sanitaria de Navarra (IdiSNA), Pamplona, Spain; Servicio de Microbiología, Hospital Universitario Son Espases, IdISBa, CIBERINFEC, Palma de Mallorca, Spain; Instituto de Investigación Sanitaria de Navarra (IdiSNA), Pamplona, Spain; Infectious Diseases Division, Clínica Universidad de Navarra, Pamplona, Spain; Servicio de Microbiología, Hospital Universitario Son Espases, IdISBa, CIBERINFEC, Palma de Mallorca, Spain; Servicio de Microbiología, Hospital Universitario Son Espases, IdISBa, CIBERINFEC, Palma de Mallorca, Spain; Service of Clinical Microbiology, Clínica Universidad de Navarra, Av. De Pio XII, 36, Pamplona, Navarre, 31008, Spain; Instituto de Investigación Sanitaria de Navarra (IdiSNA), Pamplona, Spain; Infectious Diseases Division, Clínica Universidad de Navarra, Pamplona, Spain

## Abstract

**Background:**

Few active antibiotic options are available to treat MBL-producing *Pseudomonas aeruginosa* infections, and some of these options are either poorly tolerated or have pharmacokinetic limitations. The use of aztreonam monotherapy for treating MBL-producing *P. aeruginosa* remains controversial due to the risk of selecting resistant mutants during treatment.

**Objectives:**

To describe the clinical outcomes of patients treated with ceftazidime-avibactam plus aztreonam for VIM-producing *P. aeruginosa* infections. The assessed outcomes include clinical success, clinical cure, all-cause mortality at day 28, combination therapy-associated adverse events, infection relapse and microbiological recurrence.

**Methods:**

This retrospective observational single-centre study was conducted at Clínica Universidad de Navarra, Pamplona, Spain. Eight patients with VIM-producing *P. aeruginosa* infections were included. Whole-genome sequencing of isolates was performed at Hospital Universitario Son Espases, Palma, Spain.

**Results:**

All isolates were susceptible to aztreonam and aztreonam-avibactam. No resistance mechanisms against these antibiotics were identified through whole-genome sequencing, except in one isolate that overexpressed the MexAB-OprM efflux pump. Clinical success and clinical cure were achieved in seven of eight patients, while all-cause mortality at day 28 was two of eight. Clinical cure was documented for five different infections and three distinct *P. aeruginosa* clones. No adverse events related to antibiotic therapy were reported, and no infection relapses occurred after treatment. Microbiological recurrence was observed in two cases.

**Conclusions:**

In our experience, patients with VIM-producing *P. aeruginosa* infections treated with ceftazidime-avibactam plus aztreonam mostly achieved clinical success. However, given the limited sample size, further research is required to validate these findings.

## Introduction

MBL-producing *Pseudomonas aeruginosa* (MBL-PA) poses a significant treatment challenge due to the limited number of active antibiotic options.^1^ But, even with appropriate antibiotic treatment, in-hospital mortality rates have been reported to reach as high as 38.5%.^2^

*Pseudomonas aeruginosa*-acquired MBLs confer resistance to all commercially available β-lactams alone or combined with β-lactamase inhibitors except for aztreonam and cefiderocol.^3^ However, limited data exist regarding aztreonam’s use in monotherapy against MBL-PA infections.^3^ Furthermore, aztreonam monotherapy has been associated with the selection of resistant mutants during treatment due to the overexpression of chromosomal *P. aeruginosa* AmpC β-lactamase.^4^ While aminoglycosides and colistin remain potential options, their use is reserved for dire circumstances due to their associated adverse effects, unpredictable pharmacokinetics (i.e. colistin) or poor lung penetration (i.e. aminoglycosides).^5–7^ Consequently, antibiotic options are further reduced.

Ceftazidime-avibactam in combination with aztreonam has been established as an appropriate treatment for MBL-producing *Enterobacterales*.^8,9^ This is attributed to the inability of B-type carbapenemases to hydrolyse aztreonam and the inhibitory effect of avibactam on frequently concurrent β-lactamases.^1,9^ Similarly, avibactam impairs the hydrolytic capacity of *P. aeruginosa* constitutive AmpC towards aztreonam.^3,7^ However, unlike *Enterobacterales*, *P. aeruginosa* may harbour numerous additional resistance mechanisms that confer resistance to aztreonam and avibactam. Of particular note is the overexpression of the MexAB-OprM efflux pump.^7^ Therefore, the potential benefits of this combination for treating MBL-PA infections remain a topic of ongoing debate.

This study describes the clinical outcomes of patients treated with ceftazidime-avibactam plus aztreonam for VIM-producing *P. aeruginosa* infections at our centre. The primary outcomes assessed included clinical success, clinical cure, and all-cause mortality at day 28. Combination therapy-associated adverse events, infection relapse and microbiological recurrence were also assessed.

## Methods

This retrospective study was conducted at the Clínica Universidad de Navarra, Pamplona, Spain. All adult patients diagnosed with VIM-producing *P. aeruginosa* infections who received ceftazidime-avibactam plus aztreonam for ≥72 h between January 2019 and January 2024 were included. Whole-genome sequencing of the isolates was conducted at the Hospital Universitario Son Espases, Palma, Spain.

Infections were established following the criteria set forth by the CDC (https://www.cdc.gov/nhsn/PDFs/pscManual/17pscNosInfDef_current.pdf). Ceftazidime-avibactam was administered at a dosage of 2.5 g q8h (over 2 h), while aztreonam was administered at 2 g q8h (over 30 min). Dosages were adjusted in accordance with the patient’s CL_CR_. Additional antibiotic therapy was defined as the administration of at least one additional active drug for ≥72 h. Infection source control was determined by the research team when any suspected source of infection was removed or drained.^10^ Patients were monitored for a minimum of 90 days after infection onset.

During the study period, 11 patients received treatment with ceftazidime-avibactam plus aztreonam following positive cultures for VIM-producing *P. aeruginosa*. Two patients received ceftazidime-avibactam plus aztreonam for <72 h, while one patient did not meet the infection criteria due to the bacterial count in their urine culture being below the established threshold. Consequently, the study population comprised eight patients. All included patients were consistently evaluated by the same infectious diseases team, which adhered to standardised protocols to ensure uniformity in source control measures and non-antimicrobial therapeutic strategies.

### Outcome definitions

Clinical success is defined as a composite endpoint achieved either by clinical cure or by survival on day 28 following the initial dose of ceftazidime-avibactam plus aztreonam. Clinical cure is defined as the resolution of the signs and symptoms of the infection for which ceftazidime–avibactam plus aztreonam was initiated.^11^

Microbiological recurrence was defined as the detection of the same bacteria in an infection-site culture following the completion of ceftazidime-avibactam plus aztreonam therapy. In cases where the isolate was initially detected in a sterile anatomical site (i.e. blood or cerebrospinal fluid) and an infection-site sample could not be obtained, microbiological recurrence was presumed negative if the patient achieved clinical cure.^12^ Infection relapse is defined as the reappearance of signs and symptoms consistent with the original infection and the detection of the same bacteria in an infection-site culture.^13^

### Antibiotic susceptibility

The susceptibility profiles were determined using automated microdilution (AST-427, Vitek-2, bioMérieux). The MICs of aztreonam and aztreonam-avibactam were obtained by broth microdilution following EUCAST 2024 guidelines (v.14.0). Cefiderocol MICs were determined by the aforementioned method but using UMIC cefiderocol (Bruker). Ceftazidime-avibactam, imipenem-relebactam and meropenem-vaborbactam MICs were determined using gradient diffusion strips. All tests were conducted in duplicate. Antimicrobial susceptibility tests results were interpreted in accordance with the EUCAST 2024 Clinical Breakpoints (v.14.0).

### Whole-genome sequencing

#### Library preparation

Bacterial DNA was extracted using a commercially available extraction kit (High Pure PCR Template Preparation Kit, Roche Diagnostics). Indexed paired-end libraries were prepared using the Illumina DNA Prep library preparation kit (Illumina) and then sequenced on an Illumina NovaSeq 6000 system using a NovaSeq 6000 SP Reagent Kit v1.5 (300 cycles).

#### Resistome analysis

Obtained raw reads were trimmed with Trimmomatic v0.39 and *de novo* assembled with SPAdes v3.15.5 using the –careful option. The resulting assemblies were used to infer the ST using MLST v2.23.0 (https://github.com/tseemann/mlst, accessed on 30 May 2024), to explore the presence of horizontally acquired antimicrobial resistance genes using ResFinder v4.0 (accessed on 30 May 2024), and to study the OprD porin structural integrity, as described in previous studies.^14^

In addition, a variant calling analysis was performed to examine the mutational resistome. To this end, the Snippy software v4.6.0 (https://github.com/tseemann/snippy, accessed on 30 May 2024) was employed using the *P. aeruginosa* PAO1 genome (NC_002516.2) as the reference. Single nucleotide variants (SNPs) and short insertions and deletions (InDels) identified in the *P. aeruginosa* mutational resistome genes (*n* = 40) were extracted, and natural polymorphisms were subsequently filtered.^14^

## Results

The antibiotic susceptibility profiles and whole-genome sequencing results of each isolate are presented in Table 1. All MBLs were identified as VIM-2, except for one VIM-1, which was detected along with a *bla*_CARB-4_ gene. No isolate co-produced β-lactamase capable of hydrolyzing aztreonam. Furthermore, there was no overproduction of the chromosomal AmpC nor modification of its catalytic centre, and no mutations were detected in the *ftsl* gene encoding PBP3.

**Table 1.** Antibiotic susceptibility profiles, sequence type and WGS resistome of the VIM-producing *Pseudomonas aeruginosa* isolates

Patient		MIC (mg/L)															ST	WGS resistome	
	TZP	CAZ	CZA	FEP	CLT	ATM	ATA	IMP	IMR	MEM	MVB	AMK	TOB	CIP	CST	FDC		Acquired resistome	Mutational resistome
1	≥128	16	32	≥32	≥32	**16**	**16**	≥16	>32	**8**	16	≥64	≥16	2	**2**	**0**.**25**	235	VIM-2, *aac(6’)-Ib3, aac(6’)-Il, aac(3)-I, aadA6*	*mexR* (nt424Δ11), *mexB* (S1041G, V1042A), *mexZ* (E149K), *gyrA* (T83I)
2	≥128	16	32	16	≥32	**4**	**4**	≥16	>32	16	**8**	**4**	≥16	≥4	**2**	**0**.**12**	253	VIM-2, *aac(6’)-Ib-Hangzhou*	*mexZ* (aa102-105Δ), *mexD* (S915A), *pbpA*-PBP2 (A481T), *gyrA* (T83I), *parC* (S87W)
3	≥128	16	32	16	≥32	**8**	**8**	≥16	>32	**8**	**8**	**4**	≥16	≥4	**≤0.5**	**0**.**25**	253	VIM-2, *aac(6’)-Ib-Hangzhou*	*mexZ* (aa102-105Δ), *mexD* (S915A), *pbpA*-PBP2 (A481T), *gyrA* (T83I), *parC* (S87W)
4	≥128	16	16	16	≥32	**4**	**4**	≥16	>32	**4**	**8**	**4**	≥16	≥4	**≤0.5**	**0**.**25**	253	VIM-2, *aac(6’)-Ib-Hangzhou*	*mexZ* (aa102-105Δ), *mexD* (S915A), *pbpA*-PBP2 (A481T), *gyrA* (T83I), *parC* (S87W)
5	≥128	16	32	16	≥32	**8**	**8**	≥16	>32	**8**	**8**	**4**	≥16	≥4	**≤0.5**	**0**.**25**	253	VIM-2, *aac(6’)-Ib-Hangzhou*	*mexZ* (aa102-105Δ), *mexD* (S915A), *pbpA*-PBP2 (A481T), *gyrA* (T83I), *parC* (S87W)
6	≥128	≥64	>256	≥32	≥32	**8**	**4**	≥16	>32	≥16	128	**4**	≥16	1	**2**	**0**.**5**	309	VIM-1, CARB-4, *aac(6’)-Ib3, aadA1, qnrVC1*	*mexA* (K76Q), *mexZ* (A61P), *mexE* (D533E), *oprD* (nt606insIS256-like), pbpC-PBP3a (T335S), gyrA (T83I)
7	≥128	16	16	16	≥32	**4**	**8**	≥16	>32	16	**8**	**4**	≥16	≥4	**1**	**0**.**5**	253	VIM-2, *aac(6’)-Ib-Hangzhou*	*mexZ* (aa102-105Δ), *mexD* (S915A), *pbpA*-PBP2 (A481T), *gyrA* (T83I), *parC* (S87W)
8	≥128	16	16	16	≥32	**4**	**4**	≥16	>32	**8**	**8**	**4**	≥16	≥4	**2**	**0**.**12**	253	VIM-2, *aac(6’)-Ib-Hangzhou*	*mexZ* (aa102-105Δ), *mexD* (S915A), *pbpA*-PBP2 (A481T), *gyrA* (T83I), *parC* (S87W)

AMK, amikacin; ATA, aztreonam-avibactam; ATM, aztreonam; CAZ, ceftazidime; CIP, ciprofloxacin; CST, colistin; CZA, ceftazidime-avibactam; CLT, ceftolozane-tazobactam; FEP, cefepime; FDC, cefiderocol; IMP, imipenem; IMR, imipenem-relebactam; MEM, meropenem; MVB, meropenem-vaborbactam; TOB, tobramycin; TZP, piperacillin-tazobactam.

Bold values illustrate susceptible MICs.

Isolate 1 presented a deletion in the *mexR* gene, which represses the MexAB-OprM operon. *In vitro* studies have demonstrated that any mutations that inactivate MexR result in the overexpression of the MexAB-oprM efflux pump.^15^ All isolates were susceptible to aztreonam and aztreonam-avibactam and consistent with the aforementioned findings, isolate 1 exhibited the highest MIC for these antibiotics.

A detailed account of the data associated with each patient is presented in Table 2. Clinical success and cure were achieved in seven of eight patients, while all-cause mortality on day 28 occurred in two of eight patients. Clinical cure was reported across five distinct infections and three different *P. aeruginosa* clones. No adverse events related to the antibiotic therapy were reported. No instances of infection relapse were observed among surviving patients within 90 days of infection. Furthermore, no excess mortality was recorded during this period compared with 28-day mortality. Microbiological recurrence occurred in patients 2 and 5 (2/7).

**Table 2.** Characteristics of patients with VIM-producing *Pseudomonas aeruginosa* infection and treated with ceftazidime-avibactam plus aztreonam: clinical profiles, infection features, treatment modalities and outcomes

Patient	Age, yr. (sex)	Underlying disease	CCI	Type of infection	CZA/ATM duration, days	CL_CR_, mL/min	Drainable source/drainage performed (specified)	Additional antibiotic	Clinical success/cure	Mortality at day 28 and cause	Microbiological recurrence
1	68 (M)	Cardiogenic shock secondary to AMI requiring ECMO	7	Surgical site wound infection	3^a^	33	Yes/yes (VAC replacement)	No	Yes/yes	No	No
2	74 (F)	Cholangiocarcinoma of the distal common bile duct. Treated by placement of internal-external drainage	5	Intraabdominal infection	14	155	Yes/yes (cholecystostomy and bile drainage placement)	No	Yes/yes	No	Yes
3	80 (F)	Heart failure decompensation	14	Bacteriemia	5^b^	13	No	Nebulised amikacin^b^	Yes/yes	No	No
4	50 (F)	Craniopharyngioma operated on via trans-sphenoidal route. Correction of sphenoidal fistula by flap	1	Nosocomial meningitis	14	96	No	Intranasal colistin	Yes/yes	No	No
5	73 (M)	Surgery for abdominal aortic aneurysm	7	Ventilator-associated pneumonia	6	33	No	Nebulized colistin	No/no	Death due to respiratory failure	Yes
6	64 (M)	Periprosthetic leakage micro perforation (gastroenteroanastomosis stent AXIOS^™^)	4	Intraabdominal infection	19	165	Yes/yes (ascitic drainage placement)	No	Yes/yes	No	NA
7	73 (M)	Surgical wound exudate from second kidney transplantation	7	Surgical site wound infection	18	24	Yes/yes (wound debridement and VAC placement)	No	Yes/yes	No	No
8	65 (M)	HFrEF in a patient candidate for left ventricular assist device implantation as destination therapy	5	Catheter-associated urinary infection	13^c^	62	Yes/yes (urinary catheter replacement)	No	Yes/yes	Death due to heart failure	No

AMI, acute myocardial infarction; ATM, aztreonam; CCI, Charlson comorbidity index; CL_CR_, creatinine clearance by Cockcroft-Gault equation; CZA, ceftazidime-avibactam; ECMO, extracorporeal membrane oxygenation; F, female; HFrEF, heart failure with reduced ejection fraction; M, male; NA, not available; VAC, vacuum-assisted closure.

^a^Infection onset presented with a fever spike and a purulent exudative wound. The patient received 3 days of combination therapy, after which no further exudate was observed, and the fever subsided. The antibiotic regimen was switched to ciprofloxacin, which provided coverage for isolate 1, to complete a 7-day course.

^b^The patient was admitted to the ICU due to septic shock and received five days of combination therapy. Nebulised amikacin was initially administered due to low counts of VIM-producing *P. aeruginosa* in sputum, but this was withdrawn as there was no radiological or clinical evidence of respiratory infection. Moreover, isolate 3 was detected in blood culture alongside *Enterobacter cloacae*, suggesting a probable enteric origin. Upon septic shock resolution, the antibiotic therapy was switched to aztreonam and meropenem until 2 weeks’ completion. The switch was guided by antibiogram, as isolate 3 was susceptible to meropenem.

^c^The prolonged course of antibiotic treatment was maintained because the patient was awaiting complex valvular surgery, which was delayed due to the infection. No infection-related symptoms were observed. Ultimately, the patient died from cardiac failure without infectious complications.

## Discussion

The number of reported cases of VIM-producing *P. aeruginosa* infections treated with ceftazidime-avibactam plus aztreonam remains scarce. Three cases, including osteomyelitis, tracheobronchitis and hip abscess, were found in the literature.^13,16^ In the first two cases, adjunctive treatment with amikacin and nebulised colistin was administered, while in the first and third cases, source control measures were implemented. Consistent with our findings, all cases achieved clinical resolution within 30 days. It is important to note that source control procedures were performed in five cases, which likely contributed to the high rates of clinical response.

Combination antibiotic therapy, including colistin or amikacin, has demonstrated notable *in vitro* efficacy against MBL-PA.^6,17^ However, patients with MBL-PA infections frequently present with multiple comorbidities and impaired renal function, as presented in this study (median Charlson Comorbidity Index and CL_CR_ of 6 and 47.5 mL/min). These patients are particularly susceptible to the nephrotoxic effects of these antibiotics. In our case series, no adverse events were associated with ceftazidime-avibactam plus aztreonam therapy despite the high burden of comorbidities and impaired renal function. These results are consistent with other studies suggesting that colistin should be relegated to second-line treatment of carbapenem-resistant *P. aeruginosa* when new β-lactams with β-lactamase inhibitors are suited for use.^18^

All isolates exhibited a maximum 1-fold difference in the MIC between aztreonam and aztreonam-avibactam, indicating that avibactam had no influence on enhancing aztreonam activity. However, avibactam may have contributed to the prevention of the selection of mutants that overexpress chromosomal AmpC β-lactamase. As previously stated, this mutation has been documented to emerge during monotherapy with aztreonam.^4^ Moreover, it is the most prevalent mechanism conferring resistance to classical antipseudomonal β-lactams,^3,7^ and therefore, highly likely to develop. Nevertheless, further studies must be conducted to fully elucidate avibactam’s role in preventing resistant mutant selection.

VIM-2-producing isolates exhibited ceftazidime MICs 1-fold above the susceptibility breakpoint, a phenomenon that can be attributed to the lower hydrolytic activity of VIM-2 against cephalosporins and carbapenems compared with VIM-1.^19^ As a result, ceftazidime may have enhanced antimicrobial activity against VIM-2 isolates, particularly in infection sites where drug concentrations are higher (i.e. urinary tract infections).^20^ In order to better assess the role of ceftazidime in this context, *in vitro* synergy assays considering infection-site pharmacokinetics (i.e. hollow-fibre infection models) should be performed. A similar approach should be considered for meropenem, as VIM-2-producing isolates also exhibited MICs near the susceptibility breakpoint.

Cefiderocol demonstrated potent activity against all isolates. The Phase 3 randomized studies, CREDIBLE-CR and APEKS-NP, which were conducted to evaluate the efficacy of cefiderocol, enrolled four and two patients with infections caused by MBL-PA. All patients achieved clinical cure, although one indeterminate result was observed, and there were no all-cause mortalities on day 28.^21^ While these findings are promising, it is important to acknowledge the limited patient cohort. Cefiderocol has been proposed as the recommended treatment for MBL-PA.^9^ Nevertheless, challenges such as limited accessibility in certain regions, higher MICs reported among NDM-producing isolates and resistance conferred by iron transporter mutations and other β-lactamases^22^ should prompt consideration of alternative treatments.

The present study is limited by its retrospective single-centre design, the small number of cases and the absence of a control group. A randomized clinical trial or case-control study would be the optimal design to draw definitive conclusions. Nevertheless, such studies would necessitate a considerable sample size, which is challenging in view of the rarity of VIM-producing *P. aeruginosa* infections, as evidenced by the limited number of MBL-PA infections included in cefiderocol trials.^16^ While no definitive conclusions can be drawn from the present data, it provides valuable information supporting the potential effectiveness of ceftazidime-avibactam plus aztreonam in treating these infections. To the best of our knowledge, this study represents the most significant number of VIM-producing *P. aeruginosa* infections treated with ceftazidime-avibactam plus aztreonam reported to date.

### Conclusions

In our experience, patients with VIM-producing *P. aeruginosa* infections treated with ceftazidime-avibactam plus aztreonam largely achieved clinical success and clinical cure. Additionally, the combination therapy was safe for all patients, with no infection relapse observed and a few cases of microbiological recurrence. However, further research is required to confirm these findings due to the limited sample size.
